# Trends and Prospects in Surface Engineering

**DOI:** 10.3390/ma15144839

**Published:** 2022-07-12

**Authors:** Krzysztof Rokosz

**Affiliations:** Koszalin University of Technology, Śniadeckich 2, PL 75-453 Koszalin, Poland; rokosz@tu.koszalin.pl; Tel.: +48-94-3478-354

Surface engineering is an interdisciplinary topic thatcontains many branches of science related to materials science, chemistry, and physics. At present, multidisciplinary teams are working on new materials and novel coatings with optimized mechanical, electrical, electrochemical, and antibacterial properties. Surface modification methods such as electropolishing, plasma electrolytic oxidation (micro arc oxidation), chemical and physical vapor deposition; anodic oxidation; carburization, nitrocarburization, and passivation; laser treatments and hydrothermal treatments; abrasive treatments and shot peening; as well as thermoreactive deposition and sol-gel coatings are still under the development in many laboratories all over the world. In addition, additive manufacturing technologies open up new possibilities in the production of machine elements and at the same time introduce new challenges related to surface treatment, creating new trends in the field broadly understood as surface engineering.

Seven research papers have been published in the Special Issue of Trends and Prospects in Surface Engineering. All the presented subjects are multidisciplinary, and include Zr-C coatings [1], ultraviolet laser working in cold ablation conditions for cutting labels [2], corrosion of mechanical and electrochemical polishing of steels [3], plasma electrolytic oxidation (micro arc oxidation) of lights metals [4,5,6,7].

In the paper [1] the optimal functionally graded material coating’s structure, is composed of three functional layers: (1) adhesive layer, providing high adhesion of the coating to the substrate, (2) gradient load support and crack deflection layer, improving the hardness and enhancing fracture toughness, (3) wear-resistant top layer, reducing wear, was described. In the optimisation procedure of the coating’s structure, seven decision criteria basedon the state of residual stresses and strains in the substrate/coating system were proposed. Using finite element simulations and postulated criteria, the thickness and composition gradients of the transition layer in FGM coating were determined. In order to verify the proposed optimisation procedure, Zr-C coatings with different spatial distributions of carbon concentration were produced by the Reactive Magnetron Sputtering PVD (RMS PVD) method and their anti-wear properties were assessed by scratch test and ball-on-disc tribological test.

Experimental studies aiming at the development of new technology and guidelines for shaping labels from polypropylene multilayer foil using an ultraviolet (UV) laser cutting operation in paper [2] are presented. Currently, on production lines, the shaping of labels is undertaken by mechanical cutting or laser cutting, taking into account the phenomenon of hot ablation. These technologies cause many problems such as burr formation on labels sheared edges, rapid tool wear, or heat-affected zone (HAZ) formation. The experimental tests were carried out on a specially designed laser system for cutting polypropylene foil using the phenomenon of cold ablation. Parametric analyses were conducted for several foil thicknesses t = 50, 60, 70, and 80 µm. The process parameters were optimized in terms of high efficiency and high label-cut surface quality. A new criterion has been developed for assessing the quality of UV laser cutting of polypropylene foils. The results indicate a significant effect of the cutting speed and laser frequency on the width of the degraded zone on the sheet cut edge. As a result of a developed optimization task and reverse task solution it is possible to cut labels at high speeds (v = 1.5 m/s) while maintaining a high quality of cut edge free of carbon, delamination, and color changes. A degraded zone does not exceed in the examined cases s ≤ 0.17 mm.

The effect of different polishing methods (mechanical and electrochemical) on passive layer chemistry and the corrosion behavior of stainless steels in paper [3] was presented. It was found that CrNiMo austenites have a substantially better corrosion behavior than CrMnN ones. The nickel is enriched underneath the passive layer, while manganese tends to be enriched in the passive layer. It was also noted that immersion of manganese into an electrolyte preferentially causes its dissolution. It was found that high amounts of chromium (27.4%), molybdenum (3.3%), nickel (29.4%), with the addition of manganese (2.8%) after mechanical grinding, generates a better corrosion resistance than after electrochemical polishing. This is most likely because of the introduction of phosphates and sulfates into its structure, which is known for steels with a high amount of manganese. For highly alloyed CrNiMo steels, which do not contain a high amount of manganese, the addition of phosphates and/or sulphates via the electropolishing process results in a decrease in pitting corrosion resistance, which is also observed for high manganese steels. Electropolished samples show detrimental corrosion properties when compared to mechanically polished samples. This is attributed to substantial amounts of sulfate and phosphate from the electropolishing electrolyte present onthe surface of the passive layer.

The next four papers [4,5,6,7] present the porous coatings fabrication by Plasma Electrolytic Oxidation (PEO), known also as Micro Arc Oxidation (MAO). That method is used to treat metals and alloys such as: titanium, niobium, tantalum, and their alloys. On the international market, apart from scientific works at universities, scientific research on the PEO coatings is also underway in companies such as Keronite (Great Britain), Magoxid-Coat (Germany), Mofratech (France), Machaon (Russia), as well as CeraFuse, Tagnite, Microplasmic (USA). It should also be noted that the development of the space industry and implantology will force the production of trouble-free micro- and macro-machines with very high durability. Another aspect in favor of this technique is the rate of part treatment, which does not exceed several dozen minutes, and usually only lasts a few minutes.

In position [4] the coatings enriched with zinc and copper as well as calcium or magnesium, fabricated on titanium substrate by Plasma Electrolytic Oxidation (AC-PEO) under AC conditions (two cathodic voltages, i.e., −35 or −135 V, and anodic voltage of +400 V), were presented. In all experiments, the electrolytes were based on concentrated orthophosphoric acid (85 wt%) and zinc, copper, calcium, and/or magnesium nitrates. It was found that the introduced calcium and magnesium were in the ranges 5.0–5.4 at% and 5.6–6.5 at%, respectively, while the zinc and copper amounts were in the range of 0.3–0.6 at%. Additionally, it was noted that the metals of the block S (Ca and Mg) could be incorporated into the structure about 13 times more than the metals of the transition group (Zn and Cu). The incorporated metals (from the electrolyte) into the top-layer of PEO phosphate coatings were intheir first (Cu^+^) or second (Cu^2+^, Ca^2+^ and Mg^2+^) oxidation states. The crystalline phases (TiO and Ti_3_O) were detected only in coatings fabricated at a cathodic voltage of −135 V. It has also been pointed out that fabricated porous calcium-phosphate coatings enriched with biocompatible magnesium as well as with antibacterial zinc and copper are dedicated mainly to medical applications. However, their use for other applications (e.g., catalysis and photocatalysis) after additional functionalisations is not excluded.

In the reference [5] the possible ways to fabricate advanced porous coatings that are enriched in copper on a titanium substrate through Direct Current (DC) Plasma Electrolytic Oxidation (DC-PEO) with voltage control, in electrolytes made of concentrated orthophosphoric acid with the addition of copper(II) nitrate(V) trihydrate. In these studies, solutions containing from 0 to 650 g salt per 1 dm^3^ of acid and anodic voltages from 450 V up to 650 V were used. The obtained coatings featuring variable porosity could be best defined by the three-dimensional (3D) parameter Sz, which lies in the range of 9.72 to 45.18 μm. The use of copper(II) nitrate(V) trihydrate in the electrolyte, resulted, for all cases, in the incorporation of the two oxidation forms, i.e., Cu^+^ and Cu^2+^ into the coatings. Detailed X-Ray Photoelectron Spectroscopy (XPS) studies layers allowed for stating that the percentage of copper in the surface layer of the obtained coatings was in the range of 0.24 at% to 2.59 at%. The X-Ray Diffraction (XRD) studies showed the presence of copper (α-Cu_2_P_2_O_7_, and Cu_3_(PO_4_)_2_) and titanium (TiO_2_-anatase, TiO_3_, TiP_2_O_7_, and Ti_0.73_O_0.91_) compounds in coatings.

The next step of the experiment which was performed by PEO treatments with the use of a three-phase step-up transformer with a six-diode Graetz bridge in reference [6] was shown. The voltage and the amount of salt used in the electrolyte were determined so as to obtain porous coatings. Within the framework of this study, the PEO process was carried out at a voltage of 450 VRMS in four electrolytes containing the salt as copper(II) nitrate(V) trihydrate. Moreover, we showed that the content of salt in the electrolyte needed to obtain a porous PEO coating was in the range of 300–600 g/dm^3^. After exceeding this amount of salts in the electrolyte, some inclusions on the sample surface were observed. It is worth noting that this limitation of the amount of salts in the electrolyte was not connected with the maximum solubility of copper(II) nitrate(V) trihydrate in the concentrated (85%) orthophosphoric acid. It was found that the higher the concentration of Cu(NO_3_)_2_∙3H_2_O in the electrolyte, the higher the roughness of the coatings, which may be described by 3D roughness parameters, such as Sa (1.17–1.90 μm) and Sp (7.62–13.91 μm). The thicknesses of PEO coatings obtained in the electrolyte with 300–600 g/dm^3^ Cu(NO_3_)_2_∙3H_2_O were in the range of 7.8 to 10 μm. The Cu/P ratio of the whole volume of coating measured by EDS was in the range 0.05–0.12, while the range for the top layer (measured using XPS) was 0.17–0.24. The atomic concentration of copper (0.54–0.72 at%) resulted in antibacterial and fungicidal properties in the fabricated coatings, which can be dedicated to biocompatible applications.

The last paper [7] related to PEO as a summary, the history of the PEO coatings as well as coatings enriched with phosphorus and calcium, and silver, and strontium or lanthanum, and iron or nickel, and copper, silver or zinc were described.

Some topics related to the surface engineering of metals and their alloys have been shown in theSpecial Issue. It should be pointed out that the subject is interdisciplinary and includes, inter alia, topics such as coatings, ultraviolet laser working in cold ablation conditions for cutting labels, corrosion of mechanical and electrochemical polishing of steels, as well as plasma electrolytic oxidation (micro arc oxidation) of lights metals.

The issue of surface treatment is still open for researchers, because the top surface layers, which are fabricated during physical, chemical/electrochemical, and vacuum processes, have different properties from those of treated substrates.
